# Supplementary material for: Waist Circumference and Abdominal Volume Index Can Predict Metabolic Syndrome in Adolescents, but only When the Criteria of the International Diabetes Federation are Employed for the Diagnosis
Source: Nutrients. 2019 Jun 18;11(6):1370. doi: 10.3390/nu11061370 (PMC6627132; doi:10.3390/nu11061370)
Supplement: Supplementary file 1 [file nutrients-11-01370-s001.pdf]

Supplementary Table 1. Optimal cutoff points in receiver-operator curves (ROC) analysis of different anthropometric indexes for predicting MetS in boys according to diagnostic criteria.

	IDF	Cook	de Ferranti	Weiss	Viner	Duncan	Rodríguez-Moran	Cruz & Goran	VC (%)
Fat (%)	27.0	27.5	27.3	20.2	27.6	27.3	27.3	27.3	10.2
BMI	21.4	18.5	22.8	28.9	24.0	22.8	22.8	22.8	13.6
WC	72.3	68.8	78.3	87.3	70.3	70.3	70.3	70.3	9.1
WHR	0.86	0.86	0.88	0.90	0.86	0.86	0.86	0.86	1.8
ABSI	0.07	0.08	0.08	0.08	0.08	0.08	0.08	0.08	3.7
BAI	24.8	23.5	24.8	30.7	24.6	24.8	24.8	24.8	9.3
AVI	10.5	9.6	12.3	15.4	10.7	10.6	10.6	10.6	17.1
BRI	2.57	2.52	2.52	2.52	2.52	2.50	2.50	2.50	1.0
C-Index	1.19	1.17	1.21	1.22	1.22	1.17	1.17	1.17	2.1
BAIp	20.3	24.7	24.6	24.7	24.7	24.7	24.7	24.7	6.8

Notes: ABSI, a body shape index; AVI, abdominal volume index; BAI, body adiposity index; BAIp, pediatric body adiposity index; BMI, body mass index; BRI, body roundness index; C-Index, conicity index; Fat (%), body fat percentage; VC, variation coefficient; WC, waist circumference; WHR, waist-to-hip ratio. The VC (%) was calculated as a measure of relative variability as the ratio of the standard deviation to the mean.

Supplementary Table 2. Optimal cutoff points in receiver-operator curves (ROC) analysis of different anthropometric indexes for predicting MetS in girls according to diagnostic criteria.

	IDF	Cook	de Ferranti	Weiss	Viner	Duncan	Rodríguez-Moran	Cruz & Goran	VC (%)
Fat (%)	32.4	42.7	31.9	40.9	38.6	28.3	31.9	29.8	15,8
BMI	21.9	27.9	22.1	27.9	22.3	20.3	19.4	20.2	15,0
WC	72.5	74.8	75.8	78.8	77.8	71.8	71.8	69.8	3,9
WHR	0.86	0.82	0.87	0.90	0.82	0.85	0.83	0.85	3,5
ABSI	0.07	0.07	0.07	0.07	0.07	0.07	0.07	0.07	2,2
BAI	25.7	22.0	25.7	28.9	28.3	24.9	22.0	24.7	10,7
AVI	10.9	11.0	11.4	12.6	12.2	10.3	10.9	9.9	7,3
BRI	2.86	2.98	2.98	3.91	2.50	2.53	2.53	2.53	17,4
C-Index	1.14	1.16	1.16	1.11	1.11	1.11	1.11	1.13	2,2
BAIp	21.6	28.1	21.6	26.8	26.7	21.4	21.2	21.4	13,2

Notes: ABSI, a body shape index; AVI, abdominal volume index; BAI, body adiposity index; BAIp, pediatric body adiposity index; BMI, body mass index; BRI, body roundness index; C-Index, conicity index; Fat (%), body fat percentage; VC, variation coefficient; WC, waist circumference; WHR, waist-to-hip ratio. The VC (%) was calculated as a measure of relative variability as the ratio of the standard deviation to the mean.
